# Practical Medicine; Illustrated by Cases of the Most Important Diseases

**Published:** 1843-11-25

**Authors:** 


### BIBLIOGRAPHICAL NOTICES.

*Practical Medicine; Illustrated by Cases of the Most Important Diseases.* Edited by John M. Galt, M. D. Philadelphia: Barrington & Haswell, 1843. 8vo. pp. 328.

The collection of cases of which this work is composed, are selected from the papers of the Editor's father, Dr. Alexander D. Galt, who had an "extensive practice in Williamsburg, Virginia, and the neighbouring counties, for about forty years." Dr. Galt was a pupil of the late Sir Astley Cooper. The present cases were written amid the arduous labours and distractions of country business. The chief value of the work, and which warranted its publication, are the descriptions, doubtless faithful, of the diseases of the section of country in which the author practised. Dr. Galt seems to have been a successful practitioner in the diseases mentioned in this volume—these are Intermittent and Remittent Fevers, Inflammations of the Pleura and Lungs, Inflammation of the Bronchia, Colic, Dysentery, Rheumatism, and a number of Miscellaneous Cases.

